# Targeting the Non-structural Protein 1 from Dengue Virus to a Dendritic Cell Population Confers Protective Immunity to Lethal Virus Challenge

**DOI:** 10.1371/journal.pntd.0002330

**Published:** 2013-07-18

**Authors:** Hugo R. Henriques, Eline V. Rampazo, Antonio J. S. Gonçalves, Elaine C. M. Vicentin, Jaime H. Amorim, Raquel H. Panatieri, Kelly N. S. Amorim, Marcio M. Yamamoto, Luís C. S. Ferreira, Ada M. B. Alves, Silvia B. Boscardin

**Affiliations:** 1 Laboratory of Antigen Targeting to Dendritic Cells, Department of Parasitology, University of São Paulo, São Paulo, Brazil; 2 Laboratory of Biotechnology and Physiology of Viral Infections, Oswaldo Cruz Institute, Oswaldo Cruz Foundation, Rio de Janeiro, Brazil; 3 Laboratory of Vaccine Development, Department of Microbiology, University of São Paulo, São Paulo, Brazil; 4 National Institute for Science and Technology in Vaccines, Belo Horizonte, Brazil; University of Notre Dame, United States of America

## Abstract

Dengue is the most prevalent arboviral infection, affecting millions of people every year. Attempts to control such infection are being made, and the development of a vaccine is a World Health Organization priority. Among the proteins being tested as vaccine candidates in preclinical settings is the non-structural protein 1 (NS1). In the present study, we tested the immune responses generated by targeting the NS1 protein to two different dendritic cell populations. Dendritic cells (DCs) are important antigen presenting cells, and targeting proteins to maturing DCs has proved to be an efficient means of immunization. Antigen targeting is accomplished by the use of a monoclonal antibody (mAb) directed against a DC cell surface receptor fused to the protein of interest. We used two mAbs (αDEC205 and αDCIR2) to target two distinct DC populations, expressing either DEC205 or DCIR2 endocytic receptors, respectively, in mice. The fusion mAbs were successfully produced, bound to their respective receptors, and were used to immunize BALB/c mice in the presence of polyriboinosinic: polyribocytidylic acid (poly (I:C)), as a DC maturation stimulus. We observed induction of strong anti-NS1 antibody responses and similar antigen binding affinity irrespectively of the DC population targeted. Nevertheless, the IgG1/IgG2a ratios were different between mouse groups immunized with αDEC-NS1 and αDCIR2-NS1 mAbs. When we tested the induction of cellular immune responses, the number of IFN-γ producing cells was higher in αDEC-NS1 immunized animals. In addition, mice immunized with the αDEC-NS1 mAb were significantly protected from a lethal intracranial challenge with the DENV2 NGC strain when compared to mice immunized with αDCIR2-NS1 mAb. Protection was partially mediated by CD4^+^ and CD8^+^ T cells as depletion of these populations reduced both survival and morbidity signs. We conclude that targeting the NS1 protein to the DEC205^+^ DC population with poly (I:C) opens perspectives for dengue vaccine development.

## Author Summary

Dengue is one of the most prevalent viral infections. It affects millions of people every year and can be life-threatening if left untreated. The development of a dengue vaccine is a public health priority. In the present study, we decided to use a dengue virus derived protein, named non-structural protein 1 (NS1) in an immunization protocol that targets the antigen to dendritic cells (DCs). DCs are central for the induction of immunity against pathogens and there are a few DC populations already described. NS1 was engineered in fusion with two distinct monoclonal antibodies that are capable of binding two different receptors present on the surface of these cells. NS1 targeting to one DC population (known as DEC205^+^) was able to induce anti-NS1 immune responses and confer protection to mice challenged with serotype 2 dengue virus.

## Introduction

Dengue fever is a mosquito-borne disease caused by four distinct viral serotypes (DENV1, 2, 3 and 4) [1], [2]. Over the past few decades, the alarming growth in the number of cases as well as the increase in the incidence of more serious clinical forms of the disease, the dengue hemorrhagic fever (DFH) or the dengue shock syndrome (DSS), have led the World Health Organization to prioritize the development of a dengue vaccine [1], [2]. Various formulations and vaccine antigens are currently under clinical evaluation or preclinical development [3]–[5]. Among the virus proteins that can induce protective immunity in experimental conditions is the non-structural protein 1 (NS1). NS1 is a 43–48 kDa glycoprotein expressed in infected cells and present on the cell membrane in dimeric form, but can also be secreted in dimeric and hexameric forms [6]–[8]. Anti-NS1 antibodies, which are normally detected at the beginning of a dengue infection, along with the secreted protein, are currently used in disease diagnosis [8], [9]. Anti-NS1 antibodies generated in infected individuals have been demonstrated to fix complement components leading to elimination of infected cells [10]. On the other hand, others have shown that anti-NS1 antibodies can cross react with platelets and endothelial cells and, thus, interfere with platelet aggregation and cause endothelial cell damage [11]–[13]. Despite the conflicting reports regarding the role of NS1 in the prevention of the disease, promising results were obtained with vaccine formulations containing recombinant proteins produced in bacteria [14], baculovirus [15] or encoded by DNA vaccines [16]–[18]. Different degrees of protection were observed depending on the vaccine formulation, and protective immunity seemed to be dependent on NS1-specific antibody and/or T cell responses [14], [16]–[18].

In an attempt to improve both cellular and humoral immune responses against DENV NS1, we evaluated a vaccine strategy in which the target antigen is delivered to dendritic cells (DCs). DCs are professional antigen presenting cells that link innate and adaptive immune responses [19]–[21]. An increasing amount of evidence has shown that the immunogenicity of proteins can be improved by delivering them to DCs [22]–[30]. Such specific cell targeting can be accomplished after genetic fusion of the protein to the C-terminal portion of a monoclonal antibody (mAb) with specificity for a DC surface receptor [31]. The administration of the recombinant mAb in the presence of an appropriate agonist for DC maturation increases the efficiency of antigen presentation on MHC class I and II molecules and induces T-cell help for antibody responses [23], [28], [32].

There are several DC populations in steady state [33]. Based on cell surface markers (like CD11c) and in biological functions, there are two major resident DC populations in mouse secondary lymphoid organs: the first expresses the CD8α chain and a C-type lectin endocytic receptor known as DEC205/CD205 (CD8α^+^DEC205^+^), while the second lacks the CD8α and expresses another C-type lectin receptor known as DCIR2 (CD8α^−^DCIR2^+^) [34]. CD8α^+^ and CD8α^−^ DCs seem to have different functions. For example: CD8α^+^ DCs are able to capture dying cells [35], [36] and cross-present exogenous antigens on MHC class I [37]–[39] besides being the major IL-12 producers upon activation [40]. On the other hand, CD8α^−^ DCs elicit IL-4 and more efficiently form peptide MHC II complexes [26], [34], [41].

In the last decade, the capacity of DEC205 and DCIR2 to efficiently mediate antigen presentation *in vivo* was demonstrated by injecting the corresponding anti-receptor mAbs fused to proteins derived from different pathogens [23], [25]–[28], [32], [34], [42], [43]. The delivery of vaccine proteins fused to the αDEC mAb increased the efficiency of antigen presentation and allowed protein-based vaccines to induce large numbers of Th1 type CD4^+^ T cells and antibody secreting B cells [23], [25], [27], [28], [32], [43]–[45], as well as CD4^+^ T cell dependent protection to challenge in some systems [27], [32]. Antigen targeting through the DCIR2 receptor was able to induce proliferation of CD4^+^ T cells [26], [34] and also led to robust class-switched antibody responses to T cell-dependent antigens [30]. In one case, the antibodies induced by targeting the protein to DCIR2 were able to protect mice against pneumonic plague [42].

In the present study, we generated recombinant DENV2 NS1 genetically fused to the αDEC or αDCIR2 mAbs and used them to immunize mice in the presence of the toll-like receptor (TLR) 3 and melanoma differentiation-associated gene-5 (MDA5) agonist poly (I:C), as a DC maturation stimulus. Our results show that immunization of mice with these mAbs induced high anti-NS1 antibody titers that were, at least in part, directed against conformational epitopes. Despite differences in the IgG subclass responses, we did not observe any significant difference in the affinities of the anti-NS1 antibodies in vaccinated mice. More importantly, targeting NS1 to the CD8α^+^ DC population through the DEC205 receptor induced a significantly higher number of IFN-γ producing cells and conferred protection to virus mortality and morbidity that was attributed to the generation of antigen-specific CD4^+^ and CD8^+^ T cell responses.

## Materials and Methods

### Mice

Six- to 8-week-old adult male BALB/c mice were bred under specific- pathogen free conditions at the Isogenic Mouse Facility of the Parasitology Department, University of São Paulo, Brazil. All animals were used according to the Brazilian College of Animal Experimentation (CONEP) guidelines, and the protocols were approved by the Institutional Animal Care and Use Committee (CEUA) of the University of São Paulo (protocol number 082) and by the Animal Use Ethical Committee of Fundação Oswaldo Cruz (approval ID: L-067/08).

### Plasmid generation

The cDNA encoding the DENV2 NGC strain NS1 sequence was obtained from plasmid pcENS1 [16] and cloned in frame with the carboxyl terminus of the heavy chain of mouse αDEC205 (NLDC145 clone) or αDCIR2 (33D1 clone) antibodies (kindly provided by Dr. Michel C. Nussenzweig, The Rockefeller University), as previously described [23], [28], [31], [34]. Amplification was accomplished using the Phusion High Fidelity DNA Polymerase (New England Biolabs) according to the manufacturer's instructions. The following primers were used: sense (5′-GCTCGAGGAGTTCGGTAGGTTCGATAGTGGTTGCGTTGTGAG-3′) and anti-sense (5′-GGCGGCCGCTCAGGCTGTGACCAAGGAGTTG-3′). Plasmids pDEC-NS1 and pDCIR2-NS1 were then generated and sequenced to confirm the presence of NS1 in frame.

### Expression of recombinant fusion antibodies

Plasmids containing the heavy chain of the mouse αDEC205 or αDCIR2 antibodies (pDEC-NS1, pDEC, pDCIR2-NS1 or pDCIR2) and their respective light chains (pDEC kappa or pDCIR2 kappa, kindly provided by Dr. Michel C. Nussenzweig, The Rockefeller University) were used to transform DH5α bacteria. A single bacterial clone containing each plasmid was grown for 16–18 h at 37°C under 200 rpm shaking in 200 ml of LB medium (Life Technologies) containing 100 µg/ml ampicillin (Sigma). Plasmid DNA was purified with QIAGEN Maxi Prep columns (Qiagen), according to the manufacturer's instructions.

Human embryonic kidney (HEK) 293T (ATCC No CRL-11268) cells were cultured in 150 mm plates (TPP) under standard conditions in Dulbecco's Modified Eagle's Medium (DMEM; Life Technologies) supplemented with 5% ultra-low heat inactivated fetal bovine serum (FBS, Life Technologies), 1× l-glutamine and 1× antibiotic-antimycotic (all from Life Technologies). When cell confluence reached 70–80%, FBS-supplemented DMEM was washed off and replaced by 20 mL of DMEM supplemented with 1% Nutridoma-SP (Roche), 1× l-glutamine and 1× antibiotic-antimycotic prior to polyethyleneimine (PEI)-mediated transfection with equal amounts of the corresponding heavy and light chain vector DNAs [46]. Ten µg of each vector DNA was added to a final volume of 1 mL 150 mM NaCl solution containing 4.5 µg of PEI per µg of DNA. The mix was vortexed for 10 secs, incubated for 10 min at room temperature and distributed evenly to the culture plate. Culture supernatants from 25 plates were harvested 5–6 days after transfection, cleared from cell debris by centrifugation at 1,000×g for 30 min and filtered in 0.22 µM membranes (Corning). The antibodies present in the supernatants were precipitated by addition of ammonium sulfate (Amresco) to 60% (weight∶volume) of the total culture volume. The mixture was stirred overnight at 4°C and then centrifuged at 10,000×g for 30 min at 4°C. The precipitated antibodies were resuspended in 50 mL of cold PBS (137 mM NaCl, 2.7 mM KCl, 10 mM Na_2_HPO_4_, 2 mM KH_2_PO_4_) containing 1 mM PMSF (Amresco), and dialysed against 2 L of cold PBS. Recombinant fusion antibodies were purified with Protein G beads (GE Healthcare) by incubation of 500 µl Protein G beads per 50 ml resuspended cell culture supernatants overnight at 4°C and under rotation. Supernatants were removed after centrifugation at 800×g for 10 min at 4°C. Beads were resuspended in cold PBS and transferred to a chromatography spin column (BioRad) previously equilibrated with PBS. After two rounds of washes with 5 ml cold PBS, antibodies were eluted in 5–6 fractions (500 µl each) with glycine 0.1 M (pH 3.0). Eluates were collected in tubes containing 50 µl Tris 1 M (pH 8.0) for pH neutralization. The fractions containing antibodies were pooled together and dialysed against 2 L cold PBS, filtered through 0.2 µM membranes (TPP) for sterilization and finally had their concentrations estimated by the Bradford assay (Pierce). Aliquots were then stored at −20°C until use.

### Western blots

Approximately 1 µg of each fusion antibody or the recombinant dimeric NS1 [47] were ran on 7 or 12% SDS-PAGE gels under non-reducing or reducing conditions, respectively. Gels were either stained with Coomassie Blue (Amresco) or transferred to nitrocellulose membranes (GE Healthcare). Membranes were blocked (PBS-Tween 20 0.05%, non-fat milk 5% and BSA 1%) for 1 hour at room temperature. Membranes containing non-reduced antibodies were incubated with an anti-NS1 polyclonal antibody raised against the dimeric form of NS1 [47] in a 1∶2,000 dilution. After washes with 0.05% PBS-T, membranes were incubated with horseradish peroxidase (-HRP)-anti-mouse IgG2a (1∶3,000; SouthernBiotech) for 1 h at room temperature and developed using chemiluminescence (ECL kit, GE Healthcare). Differently, membranes containing the reduced antibodies were incubated with goat anti-mouse IgG Fc specific-HRP (1∶5,000, Jackson Laboratories) plus goat anti-mouse IgG kappa-HRP (1∶3,000; SouthernBiotech). Washes and development were performed as described for the reduced membranes.

### Binding assays

Binding assays were performed using CHO cells expressing either the mouse DEC205 or the mouse DCIR2 receptors [42], kindly provided by Dr. Michel Nussenzweig (The Rockefeller University) or using freshly isolated splenocytes from naïve mice.

One hundred and fifty thousand CHO cells stably expressing either the DEC205 or the DCIR2 receptor were incubated with 10, 1 or 0.1 µg/mL of each fusion antibody on ice for 45 min. After two washes with PBS - 2% fetal bovine serum (FBS, Life Technologies), cells were incubated with anti-mouse IgG1-PE (1∶2,000) for 30 minutes on ice. After 2 additional washes, 20,000 events were acquired using FACSCalibur flow cytometer (BD Biosciences).

Additionally, each fusion mAb was tested for its capacity to bind to either CD11c^+^CD8α^+^ or CD11c^+^CD8α^−^ DCs. Splenocytes from naïve mice were harvested and suspended in PBS-FBS 2%. Five million cells were initially incubated with anti-CD16/32 (Fc block) for 15 min and then incubated with 10, 1 or 0.1 µg/mL of each fusion antibody on ice for 45 min. After 2 washes, anti-mouse IgG1-PE, anti-CD11c-APC, anti-DX5-biotin, anti-CD19-biotin, anti-MHCII-FITC and anti-CD8-PE-Cy7 were added for 45 min on ice. After another round of washes, cells were incubated with streptavidin-PerCP (BD biosciences). All mAbs were purchased from BD biosciences. Half a million events were acquired using FACS Canto flow cytometer (BD biosciences). All analyses were performed using FlowJo software (version 9.3, Tree Star, San Carlo, CA).

### Immunizations and challenge

Groups of 4–10 animals received 2 doses with a 28-day interval of 5 µg of each fusion mAb administered intraperitoneally (i.p.) together with 50 µg poly (I:C) (Invivogen). Control groups were also immunized with poly (I:C) alone or with 10 µg of recombinant NS1 protein produced as previously described [47] plus poly (I:C). Four days before or 14 days after the boost, mice were bled by retro-orbital puncture and serum was obtained. Twenty days after the boost, mice were challenged with the mouse brain adapted NGC DENV2 strain, as previously described [17]. In some experiments, immunized mice were injected i.v. with 100 µg of αCD4 (clone GK1.5) or αCD8 (clone 2.43) mAbs to deplete CD4 and CD8 T cell populations one day before the challenge. For the challenge assays, animals were anesthetized with a mixture of ketamine-xylazine and then inoculated by the intracerebral route (i.c.) with 30 µL of DENV2 NGC strain corresponding to approximately 40 LD_50_ [48]. After the challenge, mice were monitored daily for 21 days for signs of morbidity according to the following scale: 0 = no signs of paralysis or spinal cord alteration, 1 = mild paralysis in one hind leg or alteration of the spinal column with a small hump, 2 = severe paralysis in one hind leg and alteration of the spinal column with a small hump or severe paralysis in both hind legs, 3 = two severe hind leg paralysis and deformed spinal column, 4 = death [49]. After that period, animals that survived challenge were sacrificed.

### Analysis of NS1-specific antibodies

For the detection of NS1-specific antibodies, ELISA assays were performed as described previously [23]. Briefly, high-binding ELISA plates (Costar) were coated overnight with 200 ng/well refolded recombinant NS1 protein [47] in PBS. Plates were then washed 3 times with PBS-Tween 20 0.02% and blocked with PBS-Tween 20 0.02%, non-fat milk 5% and BSA 1% for 1 h at room temperature. Serial dilutions of the sera in PBS-Tween 20 0.02%, non-fat milk 5% and BSA 0.25% were incubated for 2–3 h at room temperature. The secondary antibodies used were goat anti-mouse IgG Fc-specific-HRP (1∶5,000; Jackson ImmunoResearch Laboratories) or anti-mouse IgG subclass-HRP specific antibodies (1∶3,000; SouthernBiotech). After three additional washes, plates were developed with ortho-phenylenediamine dihydrochloride (Sigma) and H_2_O_2_ as substrate and with 4 N H_2_SO_4_ as stop solution. OD_490_ was measured using a microplate reader (Biotek). Titers represent the highest serum dilution showing an OD_490_ >0.1 normalized in a log_10_ scale. The IgG1/IgG2a ratio was calculated by dividing the mean values of the highest serum dilution obtained for IgG1 by the mean value of the highest serum dilution obtained for IgG2a without normalization. Anti-NS1 antibody affinities were determined by a thiocyanate elution-based ELISA [50] as described by Rosa *et al*. [51] using pooled sera in triplicates. The percentage of binding was calculated considering the following formula: (OD_490_ in the presence of ammonium thiocyanate X 100)/(OD_490_ in the absence of ammonium thiocyanate).

### Analysis of the cellular immune response

#### Spleen cell isolation

Mice were euthanized with CO_2_ seven days after the second immunization and spleens were removed aseptically. Bulk splenocytes were obtained in suspension and washed once with RPMI 1640 (Sigma). Red blood cells were lysed with 1 mL ACK solution (150 mM NH_3_Cl, 10 mM KHCO_3_, 0.1 mM EDTA) per spleen for 2 minutes at room temperature. After two additional washes with RPMI 1640, splenocytes were resuspended in R10 (RPMI supplemented with 10% of fetal bovine serum (GIBCO), 2 mM L-glutamine (GIBCO), 10 mM Hepes (GIBCO), 1 mM sodium pyruvate (GIBCO), 1% vol/vol non-essential aminoacid solution (GIBCO), 1% vol/vol vitamin solution (GIBCO), 20 µg/mL of ciprobacter (Isofarma, Brazil) and 5×10^−5^ M 2-mercaptoetanol (GIBCO)). Cell viability was evaluated using 0.1% Trypan Blue exclusion dye and cell concentration was estimated using a hemocytometer.

#### IFN-γ ELISPOT

ELISPOT assays for the detection of IFN-γ producing splenocytes were performed using the Ready-SET-Go kit (eBioscience), according to the manufacturer's instructions. Briefly, ELISPOT plates (MAIPS; Millipore) were coated with the capture antibody overnight at 4°C. After two washes with the Coating Buffer, plates were blocked with R10 for at least 1 h at room temperature. Splenocytes were cultured for 18–20 hours at 37°C with 5% CO_2_ in the presence of 1 µg/mL recombinant NS1 protein or with the ovalbumin protein (grade II, Sigma), as a non-related protein. Plates were then washed with PBS Tween 20 0.05% and incubated with the biotinylated anti-IFN-γ antibody for 2 h at room temperature. After 4 more washes, the plates were incubated with avidin–horseradish peroxidase for 1 h at room temperature. Spots were visualized with the AEC kit (BD biosciences) and counted using an automated stereomicroscope (KS ELISPOT, Zeiss, Oberkochem, Germany). The number of IFN-γ producing cells/10^6^ splenocytes was calculated after subtracting the negative control values (number of cells in the wells containing medium only).

### Data analysis

Statistical significance (p-values) was calculated using one-way ANOVA and Tukey's honestly significantly different (HSD). Log-rank test was used to compare mouse survival times after challenge. All tests were calculated using Prism 5 software (GraphPad Software Inc, LA Jolla, CA). Differences with p≤0.05 were considered statistically significant.

## Results

### Generation of mAbs fused to NS1

**Figure 1:**
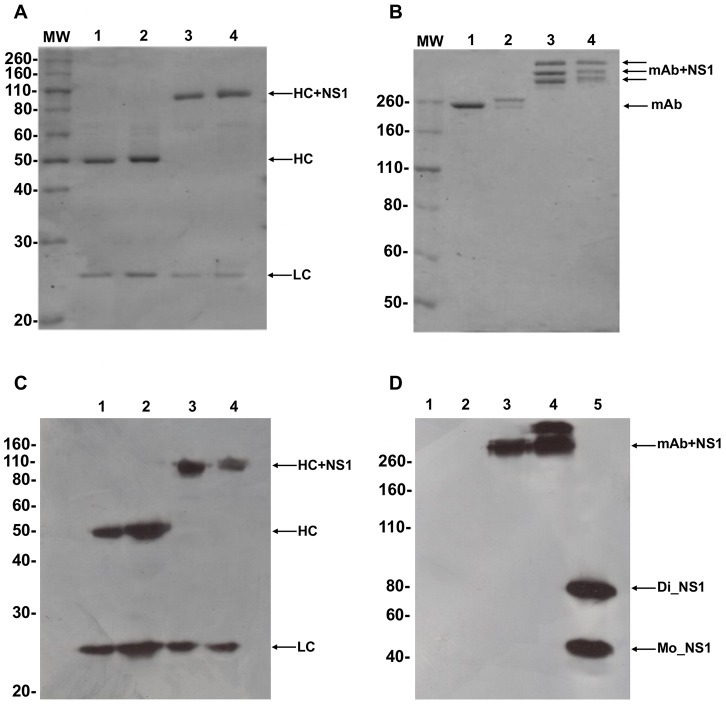
Production and characterization of αDEC-NS1 and αDCIR2-NS1 mAbs. Approximately 1 µg of each mAb was separated under reduced (A) and non-reduced (B) conditions. Both gels were stained with Coomassie Blue dye. The heavy (HC) and light (LC) chains of the following antibodies are shown: 1) αDEC, 2) αDCIR2, 3) αDEC-NS1, 4) αDCIR2-NS1. (C) Western blotting using anti-mouse IgG and anti-mouse kappa chain conjugated to HRP to verify the recognition of both heavy and light chains of each antibody. The numbers indicate the same order as in (A). (D) Western blotting on a non-reduced gel using a mouse serum raised against the dimeric form of NS1 followed by protein A conjugated to HRP. Columns 1–4, same as (A), column 5 contains 1 µg of NS1 produced in bacteria (Di_NS1: dimeric NS1 and Mo_NS1: monomeric NS1). Specific reaction was obtained only in columns containing NS1. MW, molecular weight marker in kDa.

For the generation of the fusion αDEC-NS1 or αDCIR2-NS1 mAbs, we fused the open reading frame of the sequence encoding the DENV2 NS1 protein of the NGC strain to the sequences corresponding to the C-terminal portion of αDEC or αDCIR2 heavy chains (data not shown). As controls, we also produced the αDEC and αDCIR2 antibodies without any fused antigen. All mAbs were purified and their integrity was evaluated by SDS-PAGE under non-reduced and reduced conditions (Figure 1). Figure 1A shows that heavy and light chains of the purified mAbs had the expected electrophoretic motilities (∼95 and 25 kDa, respectively, for the fusion antibodies and ∼50 and 25 kDa, for the controls) under reducing conditions. Under non-reducing conditions, the presence of multiple bands when αDEC-NS1 and αDCIR2-NS1 were separated by electrophoresis suggested the formation of multimers (Figure 1B). Figure 1C shows that specific anti-mouse antibodies for the immunoglobulin light and heavy chains recognized the recombinant mAbs. Finally, we also demonstrated (Figure 1D) that antibodies raised against DENV2 NS1 protein recognized its monomeric and dimeric forms and only the fusion mAbs, suggesting that fusion with the αDEC or αDCIR2 did not change conformational epitopes of the NS1 protein.

### The fusion mAbs bind specifically to cells expressing either DEC205 or DCIR2 receptors

**Figure 2:**
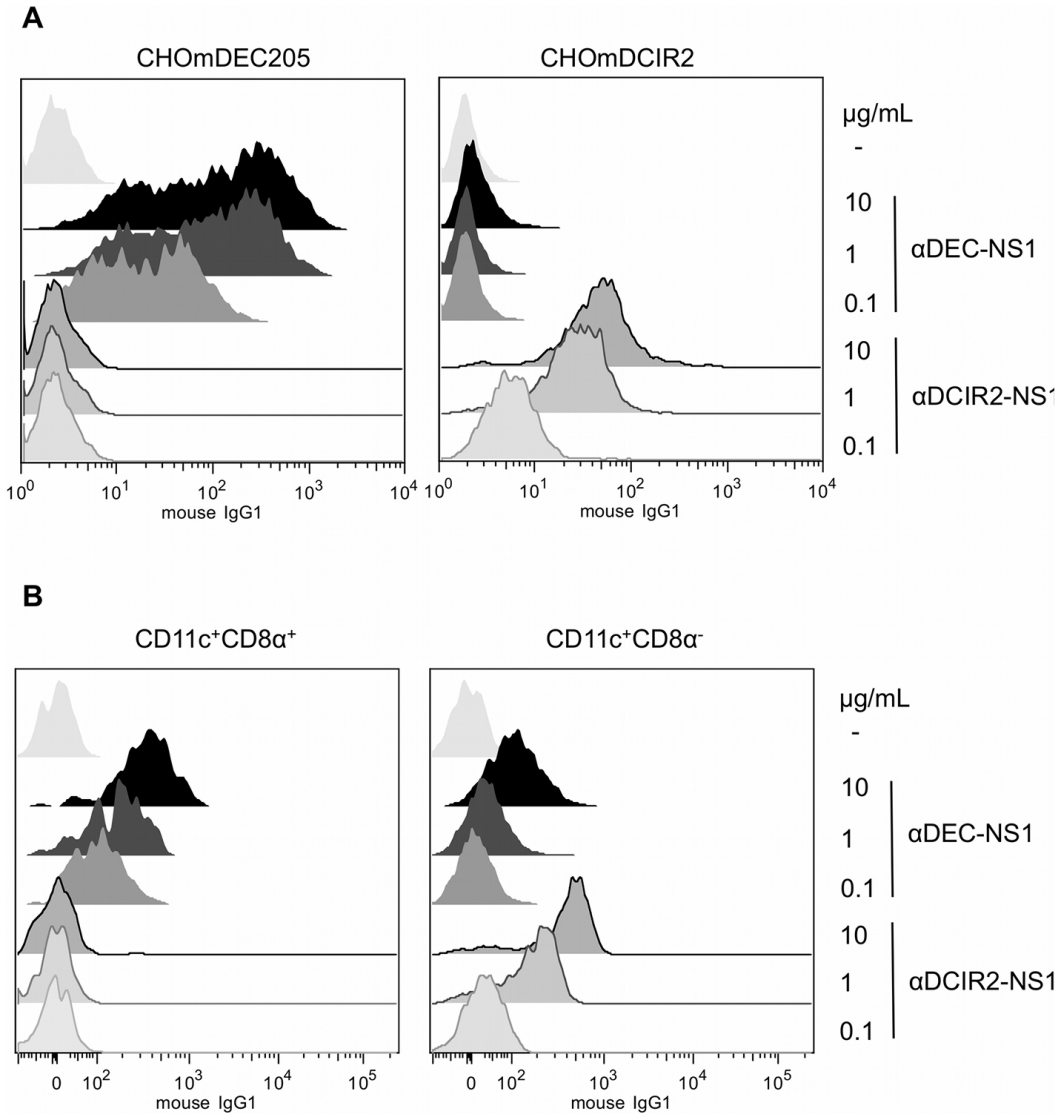
The fusion αDEC-NS1 and αDCIR2-NS1 mAbs bind to the DC DEC205 and DCIR2 receptors, respectively. (A) A density of 10^5^ CHO cells expressing either DEC205 or DCIR2 receptors were incubated on ice with 10, 1 or 0.1 µg/mL of αDEC-NS1 or αDCIR2-NS1 fusion antibodies. Binding was detected on 30.000 cells using an anti-mouse IgG1 antibody. (B) A density of 5×10^6^ splenocytes were incubated on ice with the same concentrations of the fusion antibodies described in (A). Gates on CD11c^+^CD8α^+^ (DEC205 rich population) or CD11c^+^CD8α^−^ (DCIR2 rich population) are shown in figure S1. Binding was detected on 1×10^6^ cells using an anti-mouse IgG1 antibody. One experiment representative of three is shown.

The fusion mAbs maintained their capacity to bind to their respective receptors when different concentrations (10, 1 or 0.1 µg/mL) were incubated with CHO cells expressing either DEC205 or DCIR2 receptors, or with DCs CD11c^+^CD8α^+^ or CD11c^+^CD8α^−^ (gating strategy depicted in Figure S1). Results show that the αDEC-NS1 mAb bound in a dose dependent manner to CHO cells expressing the DEC205 receptor (Figure 2A, left panel) or to CD11c^+^CD8α^+^ DCs (Figure 2B, left panel). On the other hand, the αDCIR2-NS1 mAb bound, also in a dose dependent manner, to CHO cells expressing the DCIR2 receptor (Figure 2A, right panel) and to CD11c^+^CD8α^−^ DCs (Figure 2B, right panel).

### Analysis of the anti-NS1 antibody response after the administration of two doses of the fusion mAbs in the presence of poly (I:C)

**Figure 3:**
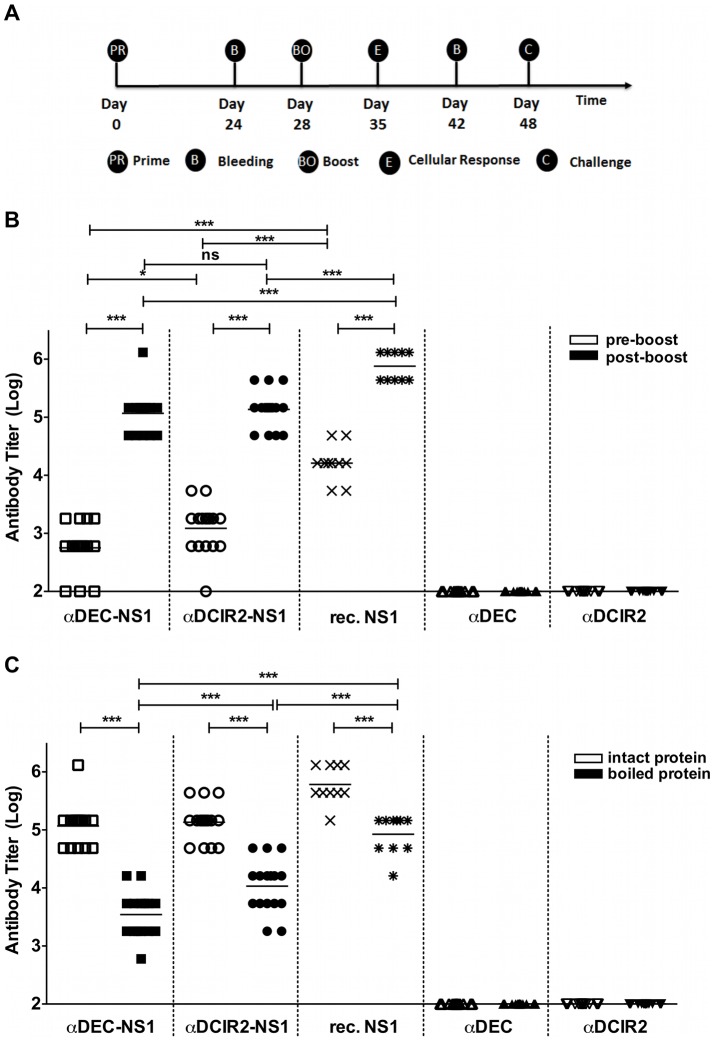
Induction of anti-NS1 antibody responses in mice immunized with the fusion αDEC-NS1 and αDCIR2-NS1 mAbs. (A) Immunization protocol describing the order of immunizations, bleedings and challenge. Mice were immunized i.p. with 2 doses of 5 µg of each fusion antibody in the presence of 50 µg of poly (I:C) or 10 µg of recombinant NS1 co-administered with 50 µg of poly (I:C). (B) Anti-NS1 antibody titers 4 days before (white symbols) or 14 days after (black symbols) the administration of the second dose. (C) Anti-NS1 antibody titers obtained 14 days after the second dose were detected using intact (white symbols) or boiled (black symbols) NS1 protein. Symbols represent individual mice and horizontal lines represent the mean value for each group. Fusion antibodies used for immunizations are depicted below. Data were analyzed by a one-way ANOVA followed by the post-test HSD Tukey. P-value indicators * and *** refer to p<0.05 and p<0.01, respectively, while ns = not significant.

BALB/c mice were immunized with two doses of each mAb (αDEC-NS1, αDCIR2-NS1, αDEC or αDCIR2) in the presence of poly (I:C), a TLR 3 and MDA5 ligand that has been used successfully in combination with other αDEC fusion antibodies [27], [42]. Poly (I:C) has been previously compared to other TLR ligands and was shown to be the most effective, at least for induction of Th1 CD4^+^ T cell responses [52]. Additionally, a group of mice received 10 µg of the recombinant NS1 protein in poly (I:C) which corresponds to 5.7 times more protein than the amount delivered in 5 µg of either αDEC-NS1 or αDCIR2-NS1. Figure 3A depicts the immunization protocol indicating when the mice were primed, boosted, bled, euthanized or challenged. The presence of anti-NS1 antibodies in the sera of the immunized animals was evaluated by ELISA, using a recombinant dimeric NS1 protein [47] 4 days before or 14 days after the administration of the booster dose (Figure 3B). Targeting the NS1 protein to either DEC205^+^ or DCIR2^+^ DC populations induced high anti-NS1 serum IgG titers particularly after the booster dose (p<0.01). However, no differences in the post boost anti-NS1 antibody titers were seen in mice immunized with the two fusion mAbs indicating that, in the presence of poly (I:C), NS1 targeting to either DEC205^+^ or DCIR2^+^ DC populations drives high antibody responses. The analysis of the primary anti-NS1 response showed significantly higher anti-NS1 titers in the mice immunized with αDCIR2-NS1 when compared to αDEC-NS1 (p<0.05). Higher anti-NS1 antibody titers were observed in mice immunized with the recombinant NS1 protein administered with poly (I:C) when compared to both αDEC-NS1 and αDCIR2-NS1 immunized groups either before (p<0.01) or after (p<0.01) the boost. In contrast, serum from mice immunized with αDEC or αDCIR2 did not recognize NS1 protein demonstrating the absence of unspecific responses.

To evaluate if the antibodies raised against NS1 were able to recognize conformational epitopes, ELISAs were performed using the boiled (heat-denatured) NS1 protein (Figure 3C). In fact, protein denaturation led to a decrease in the anti-NS1 antibody reactivity in all immunized groups (αDEC-NS1, αDCIR2-NS1 or recombinant NS1) when compared to the response obtained against the intact NS1 protein (p<0.01). This result indicated that some of the anti-NS1 antibodies generated in mice immunized with the fusion mAbs were directed against conformational epitopes. Under such conditions, mice immunized with the αDEC-NS1 showed a significantly lower response to the boiled NS1 protein than mice immunized with either the αDCIR2-NS1 (p<0.01) or recombinant NS1 (p<0.01), suggesting that the former fusion mAb induced a higher proportion of antibodies targeting conformational epitopes. In fact, the smaller reduction in the titers against the denatured protein in the animals immunized with the recombinant NS1 indicates that this immunization protocol probably induced more antibodies targeting linear epitopes. As observed in Figure 3B, no anti-NS1 titers were detected in mice immunized with the non-fused mAbs.

**Figure 4:**
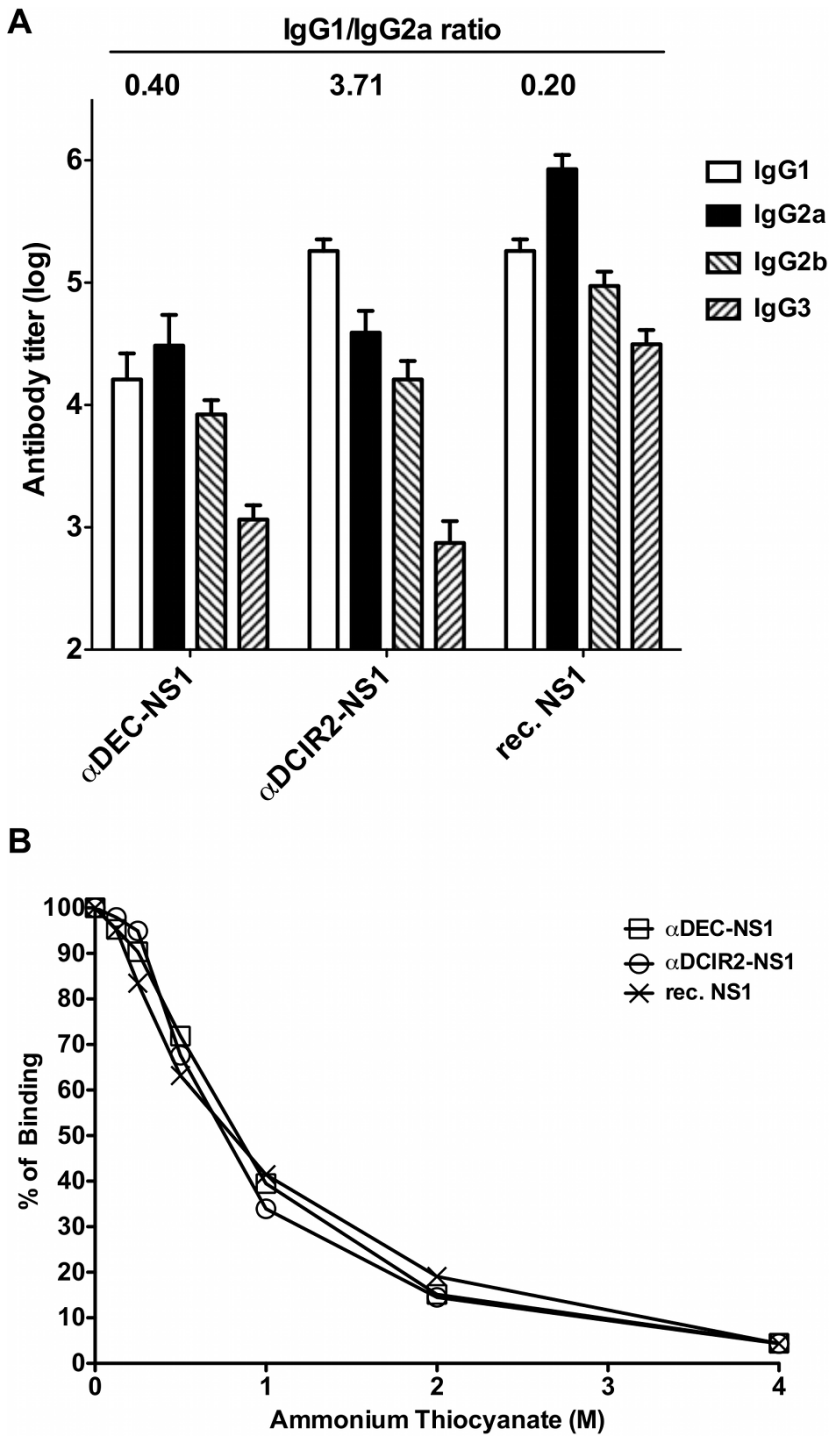
Differences in the IgG subclasses but not in the affinity of the anti-NS1 antibodies generated by the immunization with fusion mAbs or recombinant NS1 protein. Mice were immunized as described in figure 3. (A) Fourteen days after the second dose, anti-NS1 antibody titers for individual IgG subclasses were measured by ELISA. Each bar represents the mean values ± SD of the antibody titers obtained for eight mice. Numbers above the bars indicate the IgG1/IgG2a ratio obtained for each group. (B) Anti-NS1 antibodies affinity estimation by thiocyanate elution-based ELISA. Pooled sera from eight mice/group were tested 14 days after the administration of the second dose in the presence of different concentrations of ammonium thiocyanate. Total binding (100%) corresponds to the sera binding in the absence of ammonium thiocyanate. Results are expressed as the mean ± SEM of four distinct experiments performed in duplicate.

To further characterize the antibody responses, we determined the NS1-specific IgG subclass responses in vaccinated mice (Figure 4A). This analysis indicated that immunization with both fusion mAbs (αDEC-NS1 or αDCIR2-NS1) or with the recombinant NS1 protein induced antibodies of all four IgG subclasses (IgG1, IgG2a, IgG2b, and IgG3). Moreover, when the IgG1/IgG2a ratios were calculated a clear difference between the responses elicited in mice immunized with the two recombinant mAbs was observed. While an IgG1/IgG2a ratio of 0.40 was observed in mice immunized with the αDEC-NS1 mAb, a ratio of 3.71 was measured in mice immunized with the αDCIR2-NS1 mAb. These results further support the evidence that antigen targeting to different DC populations modulates the induced antibody responses [42]. Interestingly, immunization with recombinant NS1 and poly (I:C) resulted in an IgG1/IgG2a ratio similar to that observed in mice immunized with the αDEC-NS1 mAb. We cannot rule out the possibility that some of the recombinant NS1 protein is being taken up by the DEC205^+^ DCs.

We have also measured the affinity of the anti-NS1 antibodies generated in mice immunized with the fusion mAbs or with the recombinant protein. The results indicated that the anti-NS1 antibody affinity did not change regardless of the targeted DC population (Figure 4B). Also, no difference in affinity was detected between the group immunized with NS1 plus poly (I:C) and the ones immunized with both fusion mAbs (Figure 4B).

### Analysis of the cellular immune responses in mice immunized with the fusion mAbs in the presence of poly (I:C)

**Figure 5:**
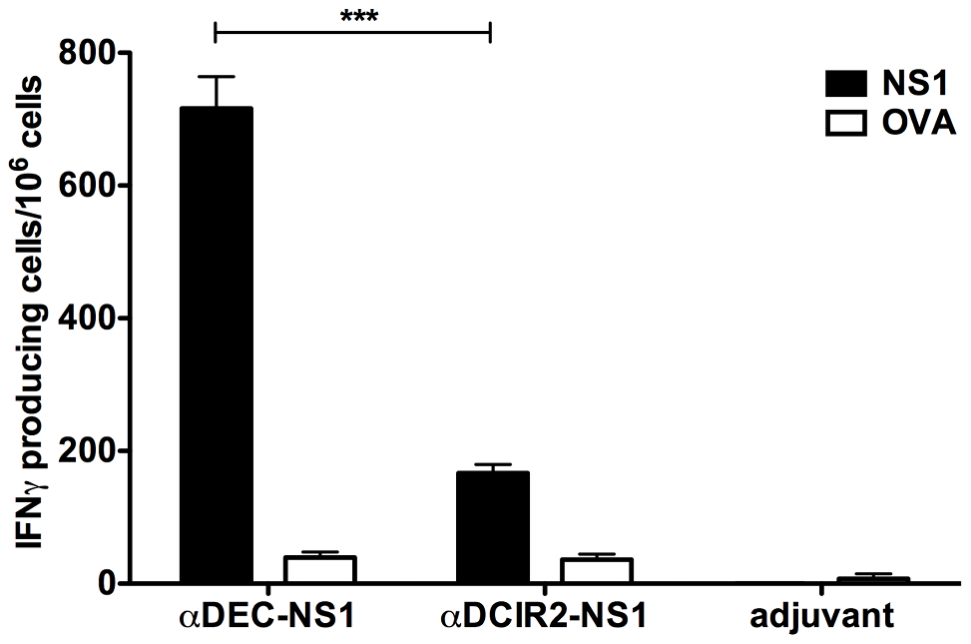
Induction of cellular immune responses in mice immunized with the recombinant αDEC-NS1 and αDCIR2-NS1 mAbs. Mice were immunized as described in figure 3 (n = 5/group). Seven days after the administration of the second dose, mice were euthanized and their splenocytes were plated in the presence or absence of 1 µg/ml of recombinant NS1 protein or ovalbumin (OVA), as a control. The number of IFN-γ producing cells was detected by ELISPOT and the bars represent the mean ± SD of pooled animals performed in triplicates. Numbers of IFN-γ producing cells in the absence of any stimulus were subtracted from the stimulated samples. Data were analyzed by a one-way ANOVA followed by the post-test HSD Tukey. *** Refers to p<0.01. Representative of 3 experiments.

The protocol described in the previous section was also used to test if the immunization with both fusion mAbs induced cellular immune responses to the DENV2 NS1. To test that possibility, we performed an ELISPOT assay to detect IFN-γ producing cells (Figure 5). Our results show that NS1 protein targeting to the DEC205^+^ DC population increases the number of NS1 specific IFN-γ producing cells when compared to the targeting to the DCIR2^+^ DC population. In contrast, negligible numbers were seen in mice immunized only with poly (I:C). This result supports previous data that, in the presence of poly (I:C), antigen targeting to the DEC205^+^ DC population increases the number of IFN-γ producing cells when compared to antigen targeting to the DCIR2^+^ population [25], [42].

### Protective anti-DENV2 responses in mice immunized with the fusion mAbs

**Figure 6:**
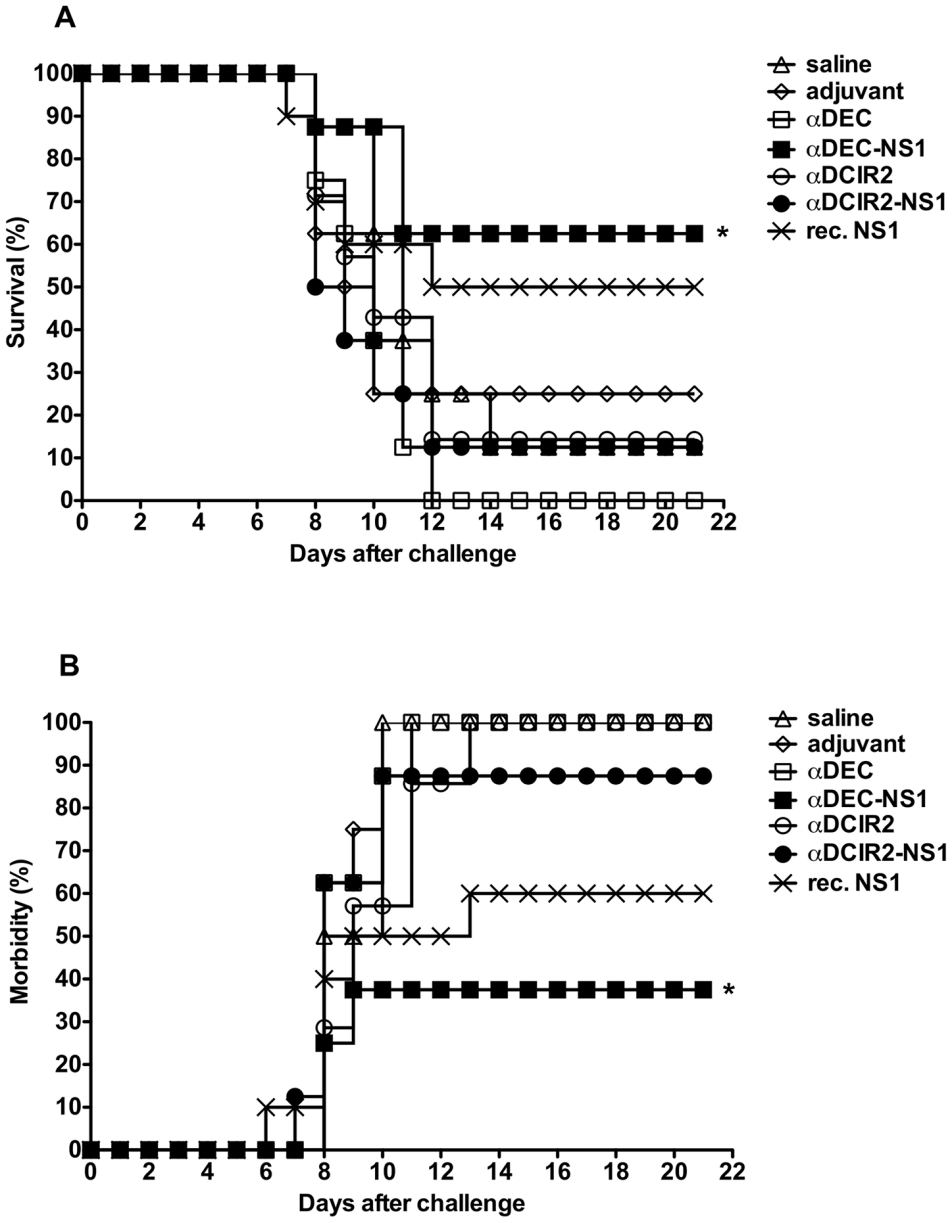
Immunization with αDEC-NS1 mAb confers partial protection to a lethal challenge with the DENV2 NGC strain. Mice were immunized as described in figure 3. Challenge was performed intracranially using 40 LD_50_ of the DENV2 NGC strain 20 days after the administration of the second dose. Survival (A) and morbidity (B) were monitored daily for 21 days after the challenge. n = 8–10 mice/group. * Refers to p<0.05 when compared to the αDCIR2-NS1, αDCIR2, αDEC and saline groups.

The protective responses elicited in immunized mice were evaluated after challenges carried out with 40 LD_50_ of the DENV2 NGC strain. As shown in Figure 6A, more than 60% of mice immunized with the αDEC-NS1 mAb survived a lethal challenge. In contrast, immunization with αDCIR2-NS1 mAb protected 12.5% of the mice. When animals were immunized with 10 µg of the recombinant NS1 and poly (I:C), 50% survived the challenge. This result is in agreement with previous results in which mice were immunized with the same amount of recombinant NS1 and another adjuvant (a genetically detoxified heat-labile toxin) [14]. In addition, a significant reduction in morbidity signs was detected in mice immunized with αDEC-NS1 mAb (37.5% of morbidity signs ×90% in the αDCIR2-NS1 mAb immunized group, p<0.05), while no difference was observed between the groups immunized with αDEC-NS1 mAb and recombinant NS1 (Figure 6B and Figure S2A). Noticeably the amount of NS1 delivered by the αDEC-NS1 mAb was 5.7 times lower than the amount of recombinant NS1 administered. In summary, targeting NS1 protein to the DEC205^+^ DC population significantly reduced mortality and morbidity associated with the virus challenge in the vaccinated mice.

**Figure 7:**
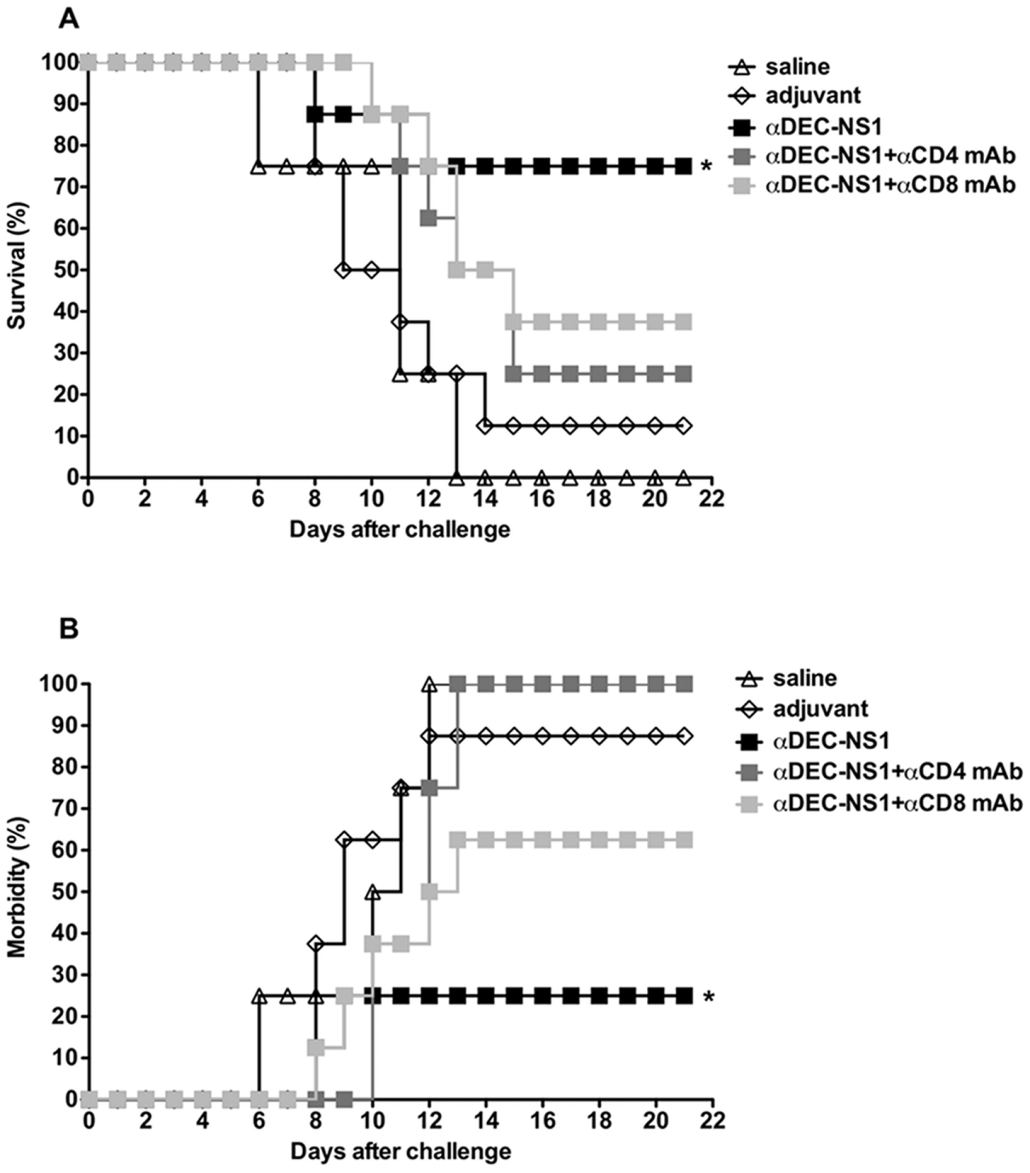
CD4^+^ and CD8^+^ T cells play important roles on the protective responses induced in mice immunized with the αDEC-NS1 mAb. Mice were immunized as described in figure 3. One day before the challenge, mice were intravenously injected with 100 µg of αCD4 or αCD8 purified antibodies to deplete the respective T cell populations. Survival (A) and morbidity (B) were monitored daily for 21 days after the challenge. n = 8 mice/group. * Refers to p<0.05 when compared to the αDEC-NS1+αCD4 mAb, adjuvant and saline groups.

To further investigate the mechanisms underlying the protective response induced in mice immunized with the αDEC-NS1 mAb, CD8^+^ and CD4^+^ T cell populations were depleted with specific anti-CD4 or anti-CD8 mAbs one day prior to the virus challenge. In an independent experiment, immunization with αDEC-NS1 mAb achieved 75% protection from death. Depletion of the CD4^+^ T cell population reduced survival to only 25% while depletion of CD8^+^ T cells reduced survival to 37.5% (Figure 7A). Depletion of CD4^+^ and CD8^+^ T cell populations also affected morbidity. All mice depleted of the CD4^+^ T cell population and 62.5% depleted of CD8^+^ T cells showed some degree of morbidity, while only 25% of the mice immunized with the αDEC-NS1 mAb presented morbidity signs (Figure 7B and Figure S2B). These results suggest that, in our model, both CD4^+^ and CD8^+^ T cells play important roles on the protective immunity induced in mice immunized with αDEC-NS1 mAb.

## Discussion

The search for an effective and safe vaccine formulation capable of preventing morbidity and mortality associated with dengue virus infection is a worldwide priority. Anti-dengue vaccine formulations based on purified recombinant proteins, although safer, are usually less immunogenic than vaccines based on attenuated or recombinant viruses. The present study demonstrated that targeting the NS1 protein to a DC population, using a genetic fusion with a mAb that binds specifically to the DEC205 receptor, promoted an enhancement of both antibody levels and cellular immune responses in mice. Furthermore, BALB/c mice immunized with two doses of the recombinant αDEC-NS1 mAb in the presence of poly (I:C) developed higher protection against a lethal challenge than the protection obtained when animals were immunized with 5.7 times more recombinant NS1 protein. This protection involved CD4^+^ and CD8^+^ T cells, as mortality and morbidity induced by the NGC DENV2 strain were increased in the absence of such cells. Altogether the present study indicates that targeting dengue virus proteins to a specific DC population represents a novel and promising alternative to achieve safer and effective anti-dengue vaccines.

Antigen targeting to DCs is an elegant approach to improve vaccine efficiency that has been intensively investigated in the last ten years. It is based on the generation of fusion antibodies that target the antigen of interest to a specific DC surface receptor [29]. Different antigens have already been used in fusion with mAbs that target different receptors. Such constructs targeted antigens from different pathogens such as HIV [25], [27], [28], Epstein-Barr virus [53], papillomavirus [54], *Plasmodium sp*. [23], [55], *Leishmania major* [26] and *Yersinia pestis* [42], [43], [45], among others. It is important to note that to accomplish specific T and B cell activation, the fusion mAbs must be administered in the presence of DC maturation stimuli or the result is tolerance [22], [23], [28], [34]. In the present study, the production of both fusion mAbs (αDEC-NS1 and αDCIR2-NS1) was accomplished successfully and resulted in the generation of antigens with preserved antigenicity and enhanced immunogenicity for the antibody-dependent and T cell-dependent (in the case of αDEC-NS1) immune responses.

The use of poly (I:C) as the adjuvant in this study was based on evidence showing that it is able to induce T and B cell immunity when administered with either αDEC 27,42,56 or αDCIR2 [42] fusion mAbs. However, the quality of the immune response seems to be modulated by the receptor used in the targeting, with the αDEC inducing a more Th1 type of response while the αDCIR2 induces multiple cytokines, including IL-4 [42]. In addition, poly (I:C) was shown to be a superior adjuvant to elicit CD4^+^ T cell immunity when used with an αDEC fusion mAb and compared to other adjuvants, such as: MALP-2 (TLR2/TLR6 ligand), Pam3Cys (TLR1/TLR2 ligand), LPS (TLR4 ligand), R-848 (TLR7/TLR8 ligand) or CpG ODN 1826 (TLR9 ligand). When antibody production against the fused antigen was considered, other adjuvants (LPS and R-848) performed similarly to poly (I:C) [52]. In our system, the use of CpG ODN 1826 with either αDEC or αDCIR2 fused to NS1 did not show significant differences in antibody responses when compared to poly (I:C), while poly (I:C) performed better in challenge experiments (unpublished data). In addition, poly (I:C) already showed a good safety record when used to induce type I interferon in cancer patients [57], and better potency as an adjuvant for responses to a protein antigen in rhesus macaques when compared to other agonists [54]. Rhesus macaques were also immunized with poly (I:C) or with poly-ICLC, a poly (I:C) analogue, which has been stabilized against serum nucleases that are present in the plasma of primates, together with αDEC fusion mAbs. Results showed induction of specific antibodies and T cell responses [55], [58].

As stated above, poly (I:C) has been mainly used together with αDEC fusion mAbs. Its use together with αDCIR2 fusion mAbs has been less documented [26], [42]. A recent paper showed a very high TLR3 expression on the surface of the CD8α^+^ DCs which also express DEC205 [59]. However, to our knowledge, such analysis has not been performed for the DCIR2^+^ DCs. So, we cannot rule out the possibility that poly (I:C) is acting directly on the DCIR2^+^ DCs nor the possibility that the poly (I:C) effect on these cells is only bystander.

One interesting characteristic of the chimeric mAbs is that the NS1 portion preserved features of the native virus protein. The significant reduction in anti-NS1 antibody titers observed when sera from immunized mice were incubated with boiled NS1 indicates that a fraction of the anti-NS1 antibodies recognized conformational epitopes, confirming that the NS1 fused to the mAbs presents a three-dimensional structure similar to the native viral protein. In fact, the results suggested that targeting NS1 to the DEC205^+^ DC population induced more anti-NS1 antibodies with specificity to conformational epitopes. Similar results were obtained when a plasmid encoding the NS1 in frame with the human tissue plasminogen activator was used in immunization protocols [16]. Under non-reducing conditions higher molecular weight bands were formed by the chimeric mAbs suggesting that multimers were also formed. It is well known that NS1 is normally found in a dimeric conformation on the surface of infected cells and forms hexamers upon secretion [60], [61]. We believe that the presence of multimers indicates that NS1 present in one antibody could also be forming complexes with another NS1 from a second antibody. Finally, to ensure that the binding of the fusion antibodies to their respective receptors was not lost by the multimer formation, we performed binding assays using either CHO cells permanently transfected with the respective receptors or freshly stained spleen derived DCs. A receptor specific and dose dependent binding was obtained for both fusion antibodies, indicating that the binding capacity of each antibody was preserved.

Immunization of mice with either αDEC-NS1 or αDCIR2-NS1 elicited high titers of anti-NS1 antibodies. This effect was detected after administration of only 2 doses (prime and boost) consisting of 5 µg of the fusion antibodies (equivalent to 1.74 µg of NS1). Administration of 10 µg of recombinant NS1 together with poly (I:C) induced higher anti-NS1 antibody titers when compared to mice immunized with the fusion mAbs. However, in this case, the amount of recombinant NS1 administered to the mice was 5.7 times higher than that administered within the fusion mAbs. Also, when NS1 was used as a DNA vaccine, antibody titers were 10 times lower [16] than those obtained with the administration of the fusion mAbs. The induction of high antibody titers was also observed in other models using antigen targeting to different DC populations [23], [30], [62]. Thus, the present results add further evidence that fusion of target antigens to the αDEC and αDCIR2 mAbs, in conjunction with poly (I:C), have a powerful effect on the generation of antigen-specific serum IgG responses far superior than other ordinarily used vaccine adjuvants.

When IgG subclasses were detected in the groups immunized with either αDEC-NS1 or αDCIR2-NS1, an approximately 10-fold difference in the IgG1/IgG2a ratio was observed. This result indicates that there are differences in the quality of the humoral immune response when these receptors are targeted in the presence of poly (I:C). Similar results were observed when antibody subclasses against LcrV protein from *Yersinia pestis* and LACK protein from *Leishmania major* were measured [26], [42]. Interestingly, the IgG1/IgG2a ratio obtained when the animals were immunized with the NS1 protein was similar to that obtained with αDEC-NS1. As already mentioned previously, we cannot rule out the possibility that some recombinant NS1 was taken up by the DEC205^+^ DC population. Furthermore, contrary to what was shown when NS1 was administered in the presence of other adjuvants such as Alum, Freund's adjuvant or detoxified heat-labile toxin [14], the IgG1/IgG2a ratio was smaller than 1 when poly (I:C) was used.

Besides humoral immunity, the contribution of T cell response was also evaluated after immunization of mice with the two chimeric mAbs. We found IFN-γ producing NS1 specific T cells mainly in mice immunized with the αDEC-NS1 mAb. The fine specificity of such cells still needs to be evaluated using peptides comprising different portions of the NS1 protein. In different models already described, the administration of fusion mAbs in the presence of DC maturation stimuli induced robust T cell proliferation [29], [63]. Also, models using transgenic T cells (either *in vitro* or transferred *in vivo*) and antigen targeting to the DEC205^+^ or DCIR2^+^ DCs showed that both DC populations may drive increases in CD4^+^ T cell proliferation [24], [26], [34].

In an attempt to verify if the induced anti-NS1 immune response would confer protective immunological status, mice were challenged with the DENV2 NGC strain. Interestingly, despite the similar IgG titers observed in the groups immunized with both fusion mAbs, partial protection and reduction in morbidity signs were only seen in the group immunized with αDEC-NS1. The recombinant NS1 immunized group showed an intermediate protection level. Of note, we cannot exclude the possibility that the DEC205^+^ DC population can process part of the recombinant NS1 protein, accounting for some degree of protection. This protection could be due to the pro-inflammatory T cell responses elicited in these animals. However, we cannot rule out the potential effect of the different IgG subclasses between the groups immunized with αDEC-NS1 and recombinant NS1. The role of CD4^+^ and CD8^+^ T cells was demonstrated when the depletion of these cell populations increased mortality and morbidity. In terms of morbidity, a more pronounced role for the CD4^+^ T cells was observed. Previous work demonstrated that antigen targeting especially to DEC205^+^ DCs provided protection in other models [28], [42], [56], [64]. However, to our knowledge, this is the first time that directing an antigen to the DEC205^+^ DCs induces CD4^+^ and CD8^+^ T cells that directly mediate protection.

Altogether the present study indicates that targeting the NS1 protein to the DEC205^+^ DC population is a promising alternative for the development of dengue vaccines based on purified recombinant proteins. Moreover, the fusion of additional dengue antigens, such as the envelope protein or immunogenic domains derived from it to the αDEC mAb may further improve the protective immunity to the virus.

## Supporting Information

Figure S1 **Gating strategy for the evaluation of binding of the fusion mAbs to the CD11c^+^CD8**α**^+^ or CD11c^+^CD8**α**^−^ DC subsets.** Splenocytes were stained on ice with different mixtures of mAbs. Doublets and CD19^+^DX5^+^ cells were excluded for further analysis. CD11c^+^MHCII^+^ were gated and separated by the expression of CD8α^+^. Analysis was performed on the CD11c^+^CD8α^+^ and CD11c^+^CD8α^−^ DCs. The numbers inside the graphs represent the percent of gated cells.
(TIF)

Figure S2 **Immunization with αDEC-NS1 mAb reduces morbidity to a lethal challenge with the DENV2 NGC strain.** Mice were immunized as described in figure 3 and challenged as described in figure 6. Signs of morbidity were recorded as described in the materials and methods section. (A) Morbidity degree obtained for each mouse on day 21 after challenge for the experiment depicted in figure 6. (B) Same as in A for experiment depicted in figure 7. n = 4–10 mice/group.
(TIF)
