# Supplementary figures and images for: Targeting the Non-structural Protein 1 from Dengue Virus to a Dendritic Cell Population Confers Protective Immunity to Lethal Virus Challenge
Source: PLoS Negl Trop Dis. 2013 Jul 18;7(7):e2330. doi: 10.1371/journal.pntd.0002330 (PMC3715404; doi:10.1371/journal.pntd.0002330)

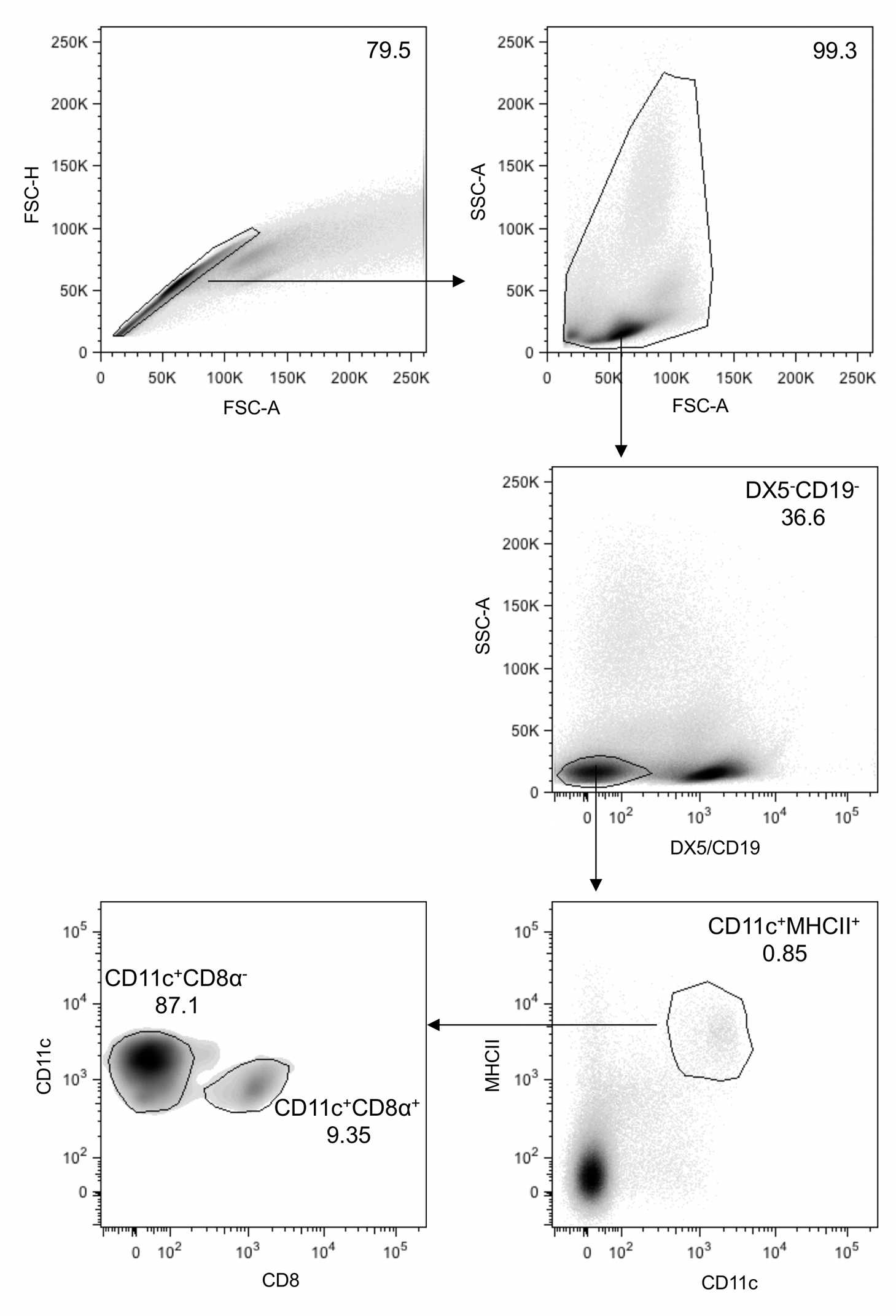

Supplement: Figure S1 — Gating strategy for the evaluation of binding of the fusion mAbs to the CD11c+CD8α+ or CD11c+CD8α− DC subsets. Splenocytes were stained on ice with different mixtures of mAbs. Doublets and CD19+DX5+ cells were excluded for further analysis. CD11c+MHCII+ were gated and separated by the expression of CD8α+. Analysis was performed on the CD11c+CD8α+ and CD11c+CD8α− DCs. The numbers inside the graphs represent the percent of gated cells. (TIF) [file pntd.0002330.s001.tif]

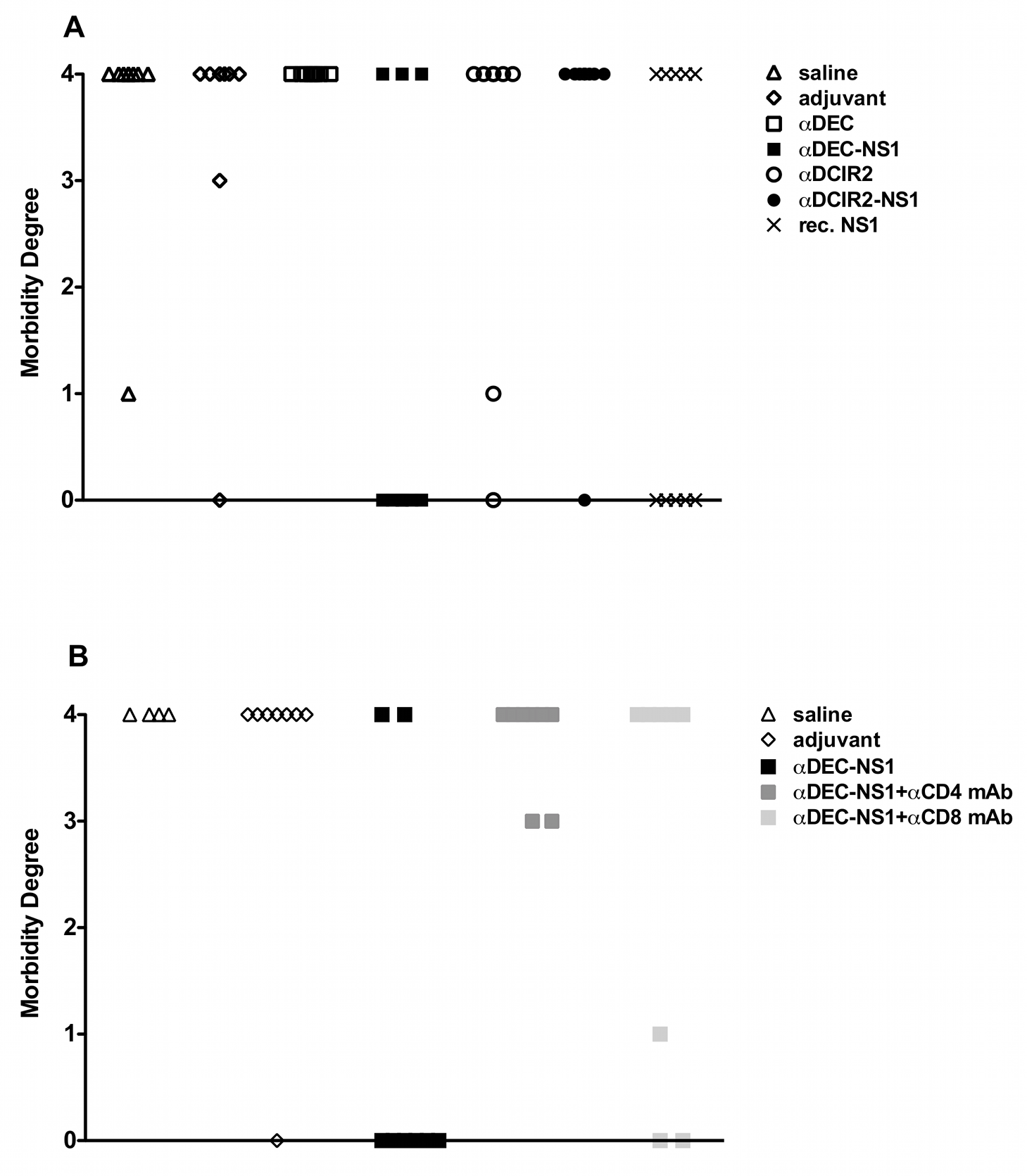

Supplement: Figure S2 — Immunization with αDEC-NS1 mAb reduces morbidity to a lethal challenge with the DENV2 NGC strain. Mice were immunized as described in figure 3 and challenged as described in figure 6. Signs of morbidity were recorded as described in the materials and methods section. (A) Morbidity degree obtained for each mouse on day 21 after challenge for the experiment depicted in figure 6. (B) Same as in A for experiment depicted in figure 7. n = 4–10 mice/group. (TIF) [file pntd.0002330.s002.tif]
